# Selection of Primer–Template Sequences That Bind with Enhanced Affinity to Vaccinia Virus E9 DNA Polymerase

**DOI:** 10.3390/v14020369

**Published:** 2022-02-10

**Authors:** Jeffrey J. DeStefano, Frédéric Iseni, Nicolas Tarbouriech

**Affiliations:** 1 Department of Cell Biology and Molecular Genetics, Bioscience Research Building, University of Maryland, College Park, MD 20742, USA; 2 Maryland Pathogen Research Institute (MPRI), University of Maryland, College Park, MD 20742, USA; 3 Unité de Virologie, Institut de Recherche Biomédicale des Armées, BP73, 91223 Brétigny-sur-Orge, France; fredericiseni@gmail.com; 4 Institut de Biologie Structurale, Université Grenoble Alpes, Centre National de la Recherche Scientifique, Commissariat à l’Energie Atomique et aux Energies Alternatives, IBS, 38000 Grenoble, France; nicolas.tarbouriech@ibs.fr

**Keywords:** vaccinia virus polymerase, E9, DNA polymerase sequence preference, SELEX

## Abstract

A modified SELEX (Systematic Evolution of Ligands by Exponential Enrichment) pr,otocol (referred to as PT SELEX) was used to select primer–template (P/T) sequences that bound to the vaccinia virus polymerase catalytic subunit (E9) with enhanced affinity. A single selected P/T sequence (referred to as E9-R5-12) bound in physiological salt conditions with an apparent equilibrium dissociation constant (K_D,app_) of 93 ± 7 nM. The dissociation rate constant (*k*_off_) and binding half-life (t_1/2_) for E9-R5-12 were 0.083 ± 0.019 min^−1^ and 8.6 ± 2.0 min, respectively. The values indicated a several-fold greater binding ability compared to controls, which bound too weakly to be accurately measured under the conditions employed. Loop-back DNA constructs with 3′-recessed termini derived from E9-R5-12 also showed enhanced binding when the hybrid region was 21 nucleotides or more. Although the sequence of E9-R5-12 matched perfectly over a 12-base-pair segment in the coding region of the virus B20 protein, there was no clear indication that this sequence plays any role in vaccinia virus biology, or a clear reason why it promotes stronger binding to E9. In addition to E9, five other polymerases (HIV-1, Moloney murine leukemia virus, and avian myeloblastosis virus reverse transcriptases (RTs), and *Taq* and Klenow DNA polymerases) have demonstrated strong sequence binding preferences for P/Ts and, in those cases, there was biological or potential evolutionary relevance. For the HIV-1 RT, sequence preferences were used to aid crystallization and study viral inhibitors. The results suggest that several other DNA polymerases may have P/T sequence preferences that could potentially be exploited in various protocols.

## 1. Introduction

The recognition of recessed 3′ termini is a hallmark of all DNA polymerases, except for some viral enzymes that can initiate synthesis using specific viral proteins (e.g., adenovirus DNA polymerase [1]). The generally accepted dogma is that both the recognition of and stable binding to the 3′ terminus are structurally driven, with the specific sequence playing little or no role in binding. However, our group has shown that viral RTs, including those from human immunodeficiency virus-1 (HIV-1), Maloney murine leukemia virus (MuLV) and avian myeloblastosis virus (AMV), bind more tightly to DNA-DNA primer–templates with runs of G (G-tract) at the 3′ primer end [2,3]. The G-tract on the DNA primer mimics the G-tract found on the polypurine tract (PPT) RNAs of these viruses. The findings suggest that RTs and PPTs may have co-evolved, leading to strong interactions and the proper orientation of the RT at the 3′ end of the PPT. Conversely, it could be argued that viral PPT sequences evolved to conform to RTs binding preferences, but played little part in RT structure and evolution. Regardless, the findings help explain why the PPT is used with higher specificity than other RNA fragments to prime second-strand DNA synthesis in retroviruses [4,5,6]. Finally, the results demonstrate a potential biological significance for DNA polymerases binding to a specific sequence with higher affinity.

It seems counterintuitive that a primer-dependent DNA polymerase (PDDP) would bind significantly more tightly to one sequence than another. Unlike structure (i.e., the presence or absence of a 3′-recessed termini) and composition (RNA or DNA), there is no clear role for sequence specificity in PDDP function. However, constraints that allow the recognition of appropriate substrates may obligate sequence specificity. That is, constraints associated with the evolution of an enzyme that can recognize and extend a recessed 3′ terminus may impart sequence preferences that are unrelated to the enzyme’s specific function [7].

Beyond RTs, we also demonstrated that *Taq* and Klenow polymerases show sequence binding preferences [7]. Interestingly, these preferences included sequences that matched the core promoter portion (3′ proximal end) of the phage T3 and T7 RNA polymerases. This suggested the intriguing possibility that phage RNA polymerases have exploited the intrinsic binding affinities of ancestral DNA polymerases to develop their promotors. Currently existing DNA polymerases may have retained the intrinsic binding preference of a common ancestor, despite there being no clear function for such a preference in DNA biology. Overall, our previous work demonstrates that PDDPs can have strong sequence biases, leading to dramatically tighter binding to specific sequences.

The above findings were uncovered using a variation of the Systematic Evolution of Ligands by Exponential Enrichment (SELEX) process [8,9] that uses a starting pool composed of ~10^14^ “random” DNA-DNA sequences in a primer–template (P/T) configuration (referred to here as “PT SELEX”). This approach allows the target polymerase to select P/Ts with sequences that it can bind with higher affinity. In this report, PT SELEX was applied to the catalytic subunit of the vaccinia virus DNA polymerase (referred to as “E9”). Protein E9 comprises 1006 amino acids and has a molecular weight of 116 kDa. It possesses 5′-3′ DNA polymerase and 3′-5′ exonuclease proofreading activities [10,11,12,13]. This protein likely functions during genomic DNA replication in a complex with its processivity factor, which is composed of two viral proteins, A20 and D4 [14,15]. In the absence of the processivity factor, E9 binds with low affinity to P/T DNA at physiological salt concentrations, but more tightly in low salt levels [16]. More recently, a high-resolution (2.7 Angstroms) crystal structure of E9 has been solved [17], although no structure in the presence of nucleic acid is available as of yet. Using PT SELEX, sequences that bind in physiological salt with significantly higher affinity were selected, demonstrating that, like RTs and *Taq* and Klenow [2,3,7], E9 can bind specific P/T sequences with enhanced affinity. Possible uses of these sequences to help understand the structure of E9 complexes with P/T are discussed.

## 2. Materials and Methods

### 2.1. Materials

Enzymes, buffers, including *Taq* polymerase and T4 polynucleotide kinase (PNK), and deoxyribonucleotides (dNTPs) were from New England BioLabs (Ipswich, MA, USA). Radiolabeled ATP (γ-^32^P) was from PerkinElmer^®^ (Waltham, MA, USA). G-25 spin columns were from Harvard Apparatus (Holliston, MA, USA). Miniprep DNA preparation kits were purchased from Qiagen (Hilden, Germany). Nitrocellulose filter disks (Protran BA 85, 0.45 μm pore size and 25 mm diameter) were from Whatman (Maidstone, UK). All DNA oligonucleotides were from Integrated DNA Technologies (IDT) (Coralville, IA, USA). The vaccinia virus E9 protein was expressed and purified as described previously [17]. All other chemicals and reagents were from Thermo Fisher Scientific (Waltham, MA, USA), Avantor™ (Radnor, PA, USA) or Sigma-Aldrich (St. Louis, MO, USA).

### 2.2. Methods

#### 2.2.1. End-Labeling of Oligonucleotides with PNK

DNA oligonucleotides were 5′-end-labeled in a 50 μL volume containing 10–250 pmol of the oligonucleotide of interest, 1X T4 PNK reaction buffer (provided by manufacturer), 10 U of T4 PNK and 5–10 μL of (γ-^32^P) ATP (3000 Ci/mmol, 10 μCi/μL). The labeling reaction was performed at 37 °C for 30–60 min according to the manufacturer’s protocol. The PNK enzyme was heat-inactivated by incubating the reaction at 75 °C for 15 min. Excess radiolabeled nucleotides (nt) were then removed via centrifugation using a Sephadex G-25 column.

#### 2.2.2. Selection of Primer–Template Sequences That Bind with Enhanced Affinity to E9 Using PT SELEX

The protocol used to select P/T sequences that bind with high affinity to E9 protein was similar to previous PT SELEX protocols, and further detail can be found in those publications [3,7]. Briefly, a starting material template containing 20 nt 5′ and 3′ fixed sequences and a 25 nt random region (i.e., 5′-GCATGAATTCCCGAAGACGC(N)_25_TCTAGAGTCGACCTGCAGGC-3′, where N is any base) was used to start the process. A 5′-^32^P-labeled primer (5′-GCCTGCAGGTCGACTCTAGA-3′) was hybridized to the template and extended with exonuclease minus Klenow polymerase. The resulting 65 nt dsDNA material was processed to produce P/T sequences with a 41 nt primer strand and a 45 nt template via digestion with Bbs I (site underlined above) which cuts the outside of its recognition site in the “N” region of the duplex. The digestion products were separated using a 12% native PAGE gel, and the radiolabeled P/T was recovered. The 3′-recessed terminus and 4 nt of 5′ single-stranded overhang were in the “N” region of the P/T. About 200 pmoles (~10^14^ different sequences) of P/T material was used in the first round of selection. The selection buffer was 20 mM Tris-HCl pH 7.5, 90 mM KCl, 60 mM NaCl, 4 mM DTT and 2% glycerol, and all selections were carried out at room temperature by incubating E9 at a ~1:10 ratio of protein to P/T with ~1 µM P/T for 30 min, before filtering the sample over nitrocellulose to capture the selected P/T sequences. Filters were washed twice with 5 mL of wash buffer (see below). The material bound to the filters was recovered with a phenol:chloroform:isoamyl alcohol extraction and ethanol precipitation, and recovery was monitored by radioactivity. Klenow polymerase (with 3′-5′ exonuclease) was used to extend the recessed 3′ terminus of the recovered material, and the blunt end products were ligated to a duplex DNA to add back the Bbs I site, essentially reproducing the dsDNA used to make the starting material (see above). A standard PCR was employed to amplify this material, and the gel-purified product was digested with Bbs I and gel-purified again to produce material for another round of selection. A notable difference in this protocol from previous ones [3,7] was the use of heparin after the 1st round of selection. For rounds 2–5, recovered material was incubated with E9 for 30 min, then heparin (1 µg/µL, ~85:1 (*w*/*w*) heparin:E9 protein) was added, and the incubation was continued for 10 min before filtering. The heparin was used to sequester the E9 molecules that were not bound to or had dissociated from P/T, and also to compete with the P/T sequences for binding to E9 (see Section 3). The PT SELEX process was stopped after 5 rounds. A limited number of recovered sequences from round 5 were sequenced by cloning and Sanger sequencing, as described previously [3,7].

#### 2.2.3. Determination of Apparent Equilibrium Dissociation Constant (K_D_,_app_) between E9 and P/T Sequences Using Nitrocellulose Filter Binding Assays

Standard reactions for K_D,app_ determinations were performed by adding 4 µL of various amounts of E9 protein diluted in buffer (20 mM Tris-HCl pH 7.5, 300 mM NaCl, 4 mM DTT, 10% glycerol) to 16 µL of a solution containing radiolabeled P/T, such that the final concentrations of components in 20 µL was: 0.1 nM P/T (5′ ^32^P end-labeled on the primer strand), 20 mM Tris-HCl pH 7.5, 90 mM KCl, 60 mM NaCl, 4 mM DTT, 0.1 µg/µL BSA and 2% glycerol. Protein was added in amounts that were in the range of the K_D,app_ value for the P/T based on preliminary experiments. Reaction components were mixed, and after 10 min at room temperature, the reactions were applied to a 25 mm nitrocellulose disk (0.45 µm pore, Protran BA 85, Whatman™) that was pre-soaked in filter wash buffer (25 mM Tris-HCl pH 7.5, 10 mM KCl). The filter was washed under vacuum with 5 mL of wash buffer at a flow rate of ~1 mL/s. Filters were then counted in a scintillation counter. A plot of bound P/T vs. E9 protein concentration was fit to the following equation for ligand binding and one-site saturation in SigmaPlot in order to determine the K_D,app_: y = B_max_(x)/(K_D_ + x), where x is the concentration of protein, y is the amount of bound aptamer and B_max_ is the amount of bound P/T at saturation.

#### 2.2.4. Dissociation Rate Constant (*k*_off_) and Half-Life (t_1/2_) Determinations

Ten nM (final concentration) 5′-^32^P end-labeled P/T was incubated for 10 min at room temperature in 72 µL of 50 nM E9 protein, 20 mM Tris-HCl pH 7.5, 90 mM KCl, 60 mM NaCl, 4 mM DTT, 0.1 µg/µL BSA and 2% glycerol. The high concentration of E9 was required due to the sequences binding relatively weakly under these conditions. At time “0”, 8 µL of heparin (10 µg/µL, ~200:1 (*w*/*w*) heparin:E9 protein) in the same buffer was added to the solution. Ten µL aliquots were removed at 0.25, 1, 2, 4, 8, 12 and 16 min and filtered over nitrocellulose, as described above. A background control was prepared by mixing 10 nM of the same 5′ ^32^P end-labeled P/T being tested with 10 µg of heparin, then adding E9 protein (50 nM final concentration) in a total of 10 ul of buffer. This tests the effectiveness of heparin at “trapping” the E9 protein and preventing its binding to P/T. This sample was incubated for 16 min before processing, and was subtracted from the other samples in the final calculations. The control sample typically showed less than 10% binding to P/T compared to the time 0.5 min sample, indicating that heparin was an effective trap at the concentration employed. The dissociation rate constant was determined by fitting the data from a plot of P/T bound to the filter vs. time to an equation for single 2-parameter exponential decay in SigmaPlot: y = ae^−bx^, where b is the dissociation rate constant (k_off_ in this case). The t_1/2_ value was determined from k_off_ using the following equation: t_1/2_ = 0.69/k_off_.

#### 2.2.5. DNA Loop-Back Extension Assays

Reactions were performed under the same conditions as the dissociation rate constant determinations, except that 25 nM of the 40 nt, 50 nt or 60 nt loop-back DNA sequences, 2 mM MgCl_2_ and 50 µM dNTPs were included. Reactions were initiated by adding E9 at 25, 50, 100 or 200 nM to a total volume of 10 µL and incubating the mixture at 30 °C for 5 min. Reactions were terminated with 10 µL of 2X loading buffer (90% formamide, 10 mM EDTA pH 8, 0.025% bromophenol blue and xylene cyanol), and the samples were run on a 16% denaturing polyacrylamide gel [18]. The material was visualized using a phosphorimager (Fuji FLA 7000).

## 3. Results

**Figure 1:**
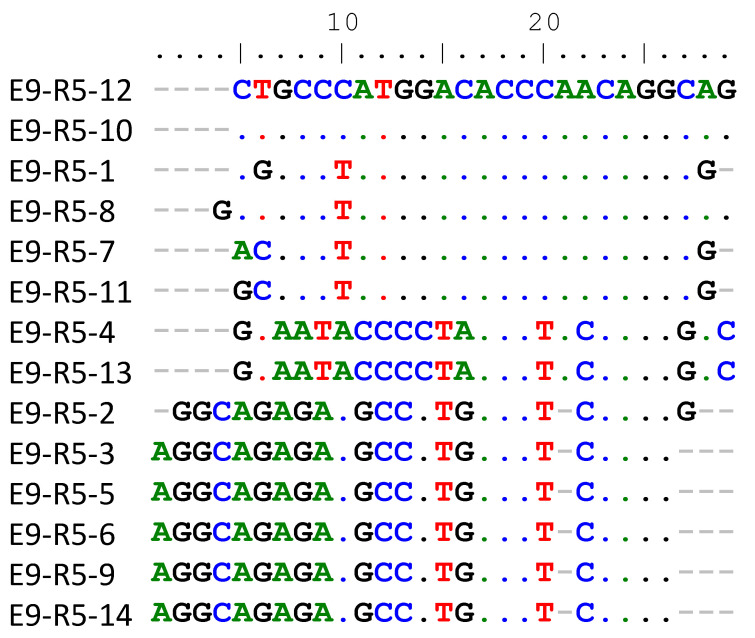
Alignment of sequences recovered from round 5 of PT SELEX with vaccinia virus protein E9. A Clustal alignment was performed using the BioEdit program with the sequence of E9-R5-12 as the reference. Only the 25 nts from the random region of the starting material are shown (sequence of primer strand, see Section 2). Sequence names indicate the protein used in the PT SELEX (E9), the SELEX round (round 5 (R5)) and the sequence number.

**Figure 2:**
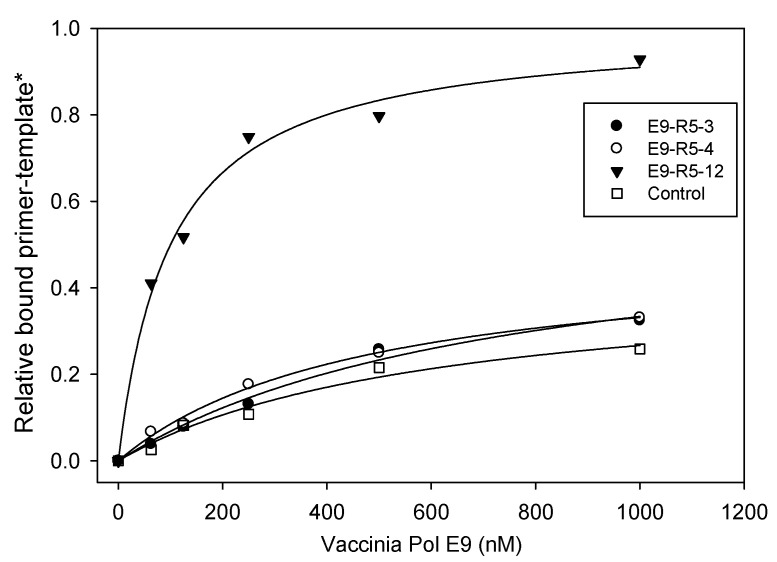
Apparent equilibrium dissociation constant (K_D,app_) analysis for material selected with E9 using PT SELEX. Nitrocellulose binding assays were used to measure binding of P/T sequences to E9 vaccinia virus polymerase, as described in Section 2. The sequences of the different P/Ts are shown in Table 1. The K_D,app_ for E9-R5-12 from this experiment was 100 nM (see Table 1). Other P/Ts did not bind well enough to determine K_D,app_ with these amounts of E9 (see Section 3). * All values were relative to the predicted amount of bound P/T at saturation (B_max_ in the equation y = B_max_(x)/(K_D_ + x) (see Section 2)) for the E9-R5-12 sample, which was set to 1. The sequences of all double stranded DNA P/Ts use in the figure are shown in Table 1.

The PT SELEX selection process used for E9 was similar to previous protocols with RTs and bacterial polymerases [3,7], with the notable exceptions of using 90 mM KCl along with 60 mM NaCl as opposed to 80 mM KCl at the start of the process, and using heparin as a competitor (see Section 2.2). Heparin is also a particularly good “trap” for polymerase, and competes strongly with the binding of the P/T, which makes it ideal for selections with polymerases [19,20]. Five rounds of selection were performed, with a step including heparin in rounds 2–5 (see Section 2). There was no increase in binding after round 4, and material from round 5 was cloned and sequenced. Fourteen sequences (shown in Figure 1) were recovered from a limited number of clones. The sequences fell into three distinct lineages, represented by E9-R5-12 (Protein-SELEX round #-sequence clone), which was recovered twice, along with four other closely related sequences, E9-R5-4, which was recovered twice, and E9-R5-3, which was recovered five times, along with one other closely related sequence (E9-R5-2). Members of each lineage, including E9-R5-12, E9-R5-4 and E9-R5-3 (all in the 41 nt primer and 45 nt template configurations), were tested for binding to E9 using the same buffer conditions that were used for selection. Stronger binding was detected only with E9-R5-12 (apparent K_D_ (K_D,app_) 93 ± 7 nM (see Table 1)). The E9-R5-4 and E9-R5-3 sequences showed low binding, similar to the starting material control sequence that was selected randomly from the starting pool (Figure 2). Binding was so low for the other sequences that a K_D,app_ could not be determined with the filter binding assay used, although this likely would have been possible if more enzyme was used, or if non-physiological lower salt concentrations were employed.

This result of low binding to the control sequence was expected because E9, in the absence of its processivity factor [14,15], binds poorly to P/T in physiological salt [16]. The low binding of sequence E9-R5-3 was somewhat unexpected, as the sequence was present five times (with one additional closely related sequence) among the 14 total recovered sequences. It is possible that it was selected for a reason not related to E9 binding (e.g., enhanced binding to the nitrocellulose filters used for selection or over-representation in the starting pool). Consistent with this, sequence E9-R5-3 also bore little resemblance to E9-R5-12 (Figure 1). Of note, another prior selection conducted without heparin produced a predominant product after eight rounds of selection that resembled E9-R5-12. The sequence of the primer strand in the random region (5′----GTAGGGTAGACAGAGCAACAG-3′) exactly matched E9-R5-12 (Table 1) over the last six nucleotides at the 3′ end, although there was no homology beyond these six nucleotides. This sequence bound just modestly better than the controls, so we elected not to continue with it. Still, this resemblance suggests that the 3′ terminal nucleotides are likely important to the observed enhanced binding. This is consistent with previous selections with RT, where the 3′ nucleotides were the main driving force for strong binding [2,3].

**Figure 3:**
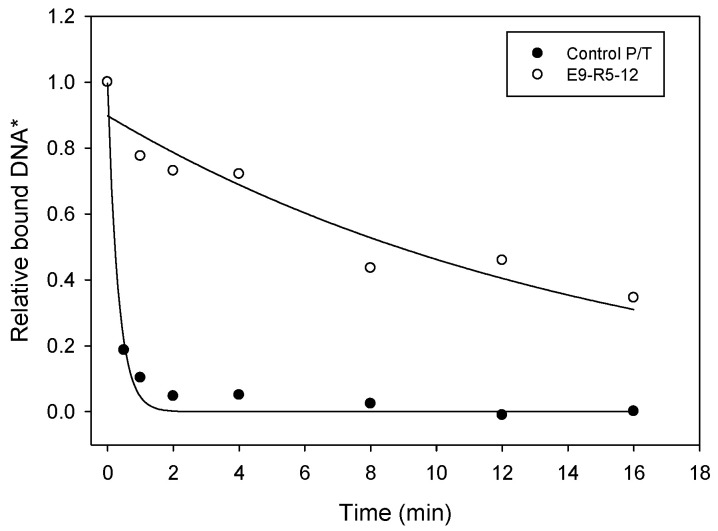
Dissociation rate constant (*k*_off_) analysis of E9-R5-12 P/T sequence. Experiments were performed using nitrocellulose filter binding, as described in Section 2. * Values are relative to the value for the amount of DNA bound to E9 at time “0” (see Section 2), which was set to “1”. The sequences of the Control P/T and E9-R5-12 double-stranded DNA sequences are shown in Table 1.

Additional experiments to examine sequence E9-R5-12 were performed using off-rate analysis rather than K_D_ analysis, as the former requires much less enzyme. E9-R5-12 bound E9 with a *k*_off_ and t_1/2_ of 0.083 ± 0.019 min^−1^ and 8.6 ± 2.0 min, respectively, while the control sequence rapidly dissociated from E9, such that *k*_off_ and t_1/2_ could not be measured by our filter binding method (Figure 3 and Table 1). A set of four modified versions of sequence E9-R5-12 were also tested for binding. The modifications replaced five nucleotides at different positions along the P/T with 5′-GACTA-3′. The first replaced the five nucleotides at the 3′ end of the primer, and the corresponding template nucleotides and the other three successively moved five nucleotides toward the 5′ end of the primer. A *k*_off_ was not measurable with any of these modifications (Table 1). This suggests that changes along most of the primer–template can affect binding. This result was different from a previous result with HIV RT, where the nucleotides near the 3′ primer terminus were the only ones required for tight binding [2].

**Figure 4:**
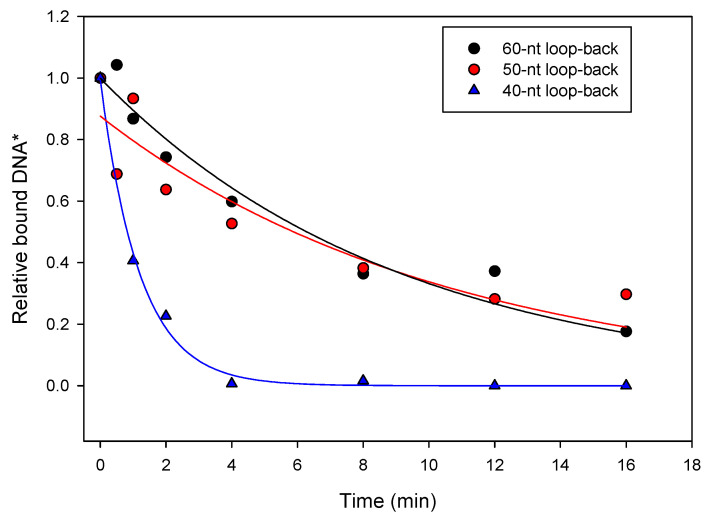
Dissociation rate constant (*k*_off_) analysis of loop-back sequences derived from the E9-R5-12 P/T sequence. Loop-backs are described in Section 3 and shown in Table 1. Experiments were performed using nitrocellulose filter binding, as described in Section 2. * Values are relative to the value for the amount of loop-back DNA bound to E9 at time “0” (see Section 2), which was set to “1”.

Previously, single-stranded loop-back DNA primer–templates were used to examine the length requirements for the tight binding of HIV RT to selected primer–templates [21]. We found that adding one extra nucleotide to the 5′ overhang, increasing it from four to five nucleotides, modestly improved binding. Therefore, three loop-back primer–template structures were prepared using the E9-R5-12 sequence. They comprised 60, 50 and 40 nucleotides with five nucleotide 5′ overhangs, three nucleotide loops and hybrid regions of 26, 21 and 16 base pairs (Table 1). Measurements of *k*_off_ indicated that the 60 and 50 nucleotide structures bound to E9 with essentially the same stability as the original E9-R5-12 sequence (Table 1). This indicated that adding an additional nucleotide to the overhang did not significantly affect binding. In contrast, the 40 nucleotide loop-back bound much less stably (Figure 4 and Table 1). Although the results at first glance suggest that stable binding requires a duplex region longer than the 16 nt in the 40 nt loopback, the 40 nt loop-back is also missing nucleotides in the duplex region that may be critical for strong binding. Results in Table 1 show that nucleotide changes within the 20 nt duplex region proximal to the 3′ primer end all result in a loss of strong binding. The 40 nt loop-back construct contains only 16 of the 20 nucleotides. Therefore, it is not clear if the loss of strong binding resulted from the shortened duplex region or is related to specific sequence requirements.

**Figure 5:**
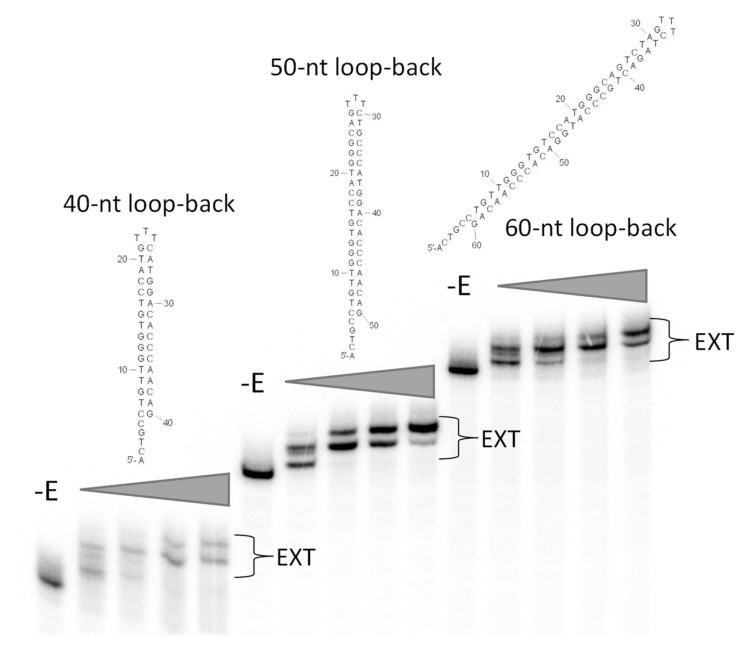
Extension of radiolabeled loop-back DNA with E9 vaccinia virus polymerase. Twenty-five nM of the indicated loop-back DNA was labeled at the 5′ end with ^32^P and extended with increasing amounts of E9 polymerase (25 nM, 50 nM, 100 nM or 200 nM) for 5 min at 30 °C, as described in Section 2. Positions of extended products are indicated, while the unextended DNA is shown in the –E (no enzyme) lane. The sequences of all loop-back DNAs use in the figure are also shown in Table 1.

To verify that E9 bound to the loop-back structures in a productive orientation, the loop-backs were 5′-end-labeled with ^32^P and used in primer extension reactions (Figure 5). All the loop-back structures were extended by E9, even the 40 nucleotide loop-back that bound more weakly to the polymerase. The extension was clearly distributive rather than processive, as the 5 nt addition required to reach the end of the template occurred progressively with shorter products present with lower amounts of enzyme. This is consistent with the distributive nature of E9 in the absence of its processivity factor in physiological salt [16].

Previously selected P/T sequences from RTs [2,3] and *Taq* and Klenow polymerases [7] contained biologically relevant sequences (resembling the PPT in the case of RTs), or sequence regions with homology to phage RNA polymerase promoter sequences (for *Taq* and Klenow). We searched for E9-R5-12-related sequences in the enriched results for RTs, *Taq* and Klenow, but could not find any resemblance. To further examine possible relationships between E9-R5-12 and previously recovered tight-binding P/Ts to other polymerases, we conducted binding dissociation and primer extension experiments using E9 (Supplemental Materials. Primer–templates selected with PT SELEX for HIV RT, *Taq* and Klenow dissociated rapidly from E9 (Table S1 and Figure S1). Like the control shown in Figure 3, the dissociation was too fast to determine a dissociation constant using filter binding. Interestingly, all the P/Ts were extended in primer extension reactions, and there was no clear advantage for the extension of the E9-R5-12 sequences compared to the others (Figure S2). This was synonymous to the experiment with the 40 nt loop-back in Figure 5, which, despite binding less stably to E9 (Figure 4), was extended similarly to the 50 and 60 nt loop-backs (Figure 5). We should note that the two assays use different reaction conditions, with the primer extension assays containing Mg^2+^ and dNTPs that are not included in the binding dissociation assays (see Section 2.2). These could have affected binding to E9. Magnesium could not be added to the binding assays, as it would activate the 3′-5′ exonuclease activity of E9, leading to erroneous measurements.

Curiously, E9-R5-12 contains 5′-CCCAT-3′ and 5′-CCCAA-3′ motifs that are separated by five nucleotides on the primer strand. These are similar to the well-characterized “CCAAT” box (also referred to as the “CAT” or “CAAT” box), present upstream of many eukaryotic promoters, that is a binding site for transcription factors [22]. However, the function of these sequences in E9 binding was not explored. There was a notable match to a region of poxvirus genomes. Sequence E9-R5-12 matches with complete homology over a 12 nucleotide region (5′-CATGGACACCCA-3′ on the primer strand) in the coding region of the B20R gene from some poxviruses. In this case, the primer strand of E9-R5-12 matches the minus strand of the poxvirus gene, and the template strand matches the plus strand. There is very little known about the protein encoded by the B20R gene. One report suggests that the gene may be involved in interferon evasion [23]. The 12 nucleotide sequence is close to the right end of the genome, but not in the putative regulatory region, and it is not near the putative start of virus replication, as described in [24]. Although the chances of a 12 nucleotide exact match in a typical poxvirus genome are low (the sequence would be expected to be present one time over 16,777,216 nucleotides, with about a 1/40 chance of being present in a ~200,000 base-pair (i.e., 400,000 nucleotides) poxvirus genome), there is no clear reason to believe there is any biological significance, especially since the segment is in the coding region of only a signal viral gene.

## 4. Discussion

In this report, we show that E9, like other DNA polymerases [2,3,7], demonstrates preferential binding to specific P/T sequences. Unlike previous P/T sequences selected for other polymerases, there was no clear biological significance for the selected sequence, and the selected sequence did not share homology with those selected with other polymerases. However, it is important to point out that only a few P/T sequences have been selected for preferential binding to polymerases [2,3,7], and it is possible that the E9 sequence could be related to other sequences that bind with high affinity to polymerases.

With respect to binding affinity, the selected E9-R5-12 P/T sequence, though binding much more tightly to E9 than controls, still bound with a K_D_ that was more than an order of magnitude higher when compared to sequences selected for tight binding to RTs and *Taq* and was more comparable to binding with Klenow [7]. The weak binding suggests that preferred sequences cannot overcome the generally weak binding of E9 to P/Ts in physiological salt [16]. The enzyme demonstrates distributive synthesis under these conditions but can be switched to a processive mode in the presence of its processivity factor or in low salt [14,15,16]. This suggests that high-affinity binding, which is presumably required for the completion of DNA synthesis over the long viral genome, is dependent on association with the processivity factor. Interestingly, P/T sequences selected for binding to Klenow also bound relatively weakly. Klenow is derived from *E. coli* Pol I, an enzyme involved in DNA synthesis repair. Like E9 in the absence of the processivity factor, Pol I also demonstrates distributive synthesis [25], which may be beneficial for a repair enzyme that typically functions over short DNA segments.

We were unable to determine the features of the selected E9-R5-12 P/T sequence that were responsible for tight binding. Progressively replacing five nucleotide segments in the P/T hybrid region abrogated the tight binding to E9 in all cases. This suggests that strong binding requires sequences present throughout the duplex. A more precise examination of the effects of individual nucleotides in the selected P/T on binding was not undertaken, as the five nucleotide replacement approach did not allow us to home in on a particular region. With HIV RT, only the G-tract sequences near the 3′ primer terminus were required for strong binding, and this was the region of the selected P/T sequence that matched the HIV PPT region [2]. If this were the case for E9, the P/Ts containing the 3′ proximal 10–15 nucleotides (Table 1) would have been expected to bind like E9-R5-12, but they did not. In a subsequent report, it was shown the G-tract likely induces a bend in the P/T that may allow it to fit better into the RT active site [26]. HIV RT and other polymerases typically induce bends in P/T sequences upon binding. The pre-bent nature of the select HIV RT P/T sequences may have induced strong binding by reducing the energy required for bending and maintaining the bend of the bound P/T. It would be interesting to examine the E9-R5-12 sequence in the future to see if it is also bent. Since there is not a current crystal structure of E9 bound to DNA P/T, the extent to which it bends the P/T during binding is not known.

With HIV RT, a 38 nucleotide loop-back structure (15 nucleotides in the duplex region) that retained tight binding was produced based on the selected P/T sequence [21]. For E9, a similar 40 nucleotide structure (16 nucleotides in the duplex region) did not retain tight binding, while a 50 nucleotide loop-back (21 nucleotides in the duplex region) did (Figure 5 and Table 1). This suggests that the two enzymes require a similar length of minimal duplex for strong binding. However, the E9 results are less clear because the 40 nt loop-back sequence, which showed low binding, was also missing specific sequences that may have been required for tight binding (see Section 3). The binding affinity of the HIV 38 nucleotide loop-back was improved by placing two O-CH_3_ groups at the 2′ sugar position of the -2 and -4 (relative to the 3′ terminal primer base) positions in the template strand [27]. This allowed the crystallization (at 2.3 Angstroms) of the loop-back with HIV RT in the absence of any cross-linking agent [28]. This approach has been used in subsequent reports analyzing various drugs and mutations [29,30,31,32], and these are the only reported structures of HIV RT with nucleic acid in the absence of cross-linking. Since the E9 loop-backs bind about two to three orders of magnitude less tightly to E9 than the O-CH_3_-modified HIV RT 38 nucleotide loop-back, it is likely that additional modification (e.g., O-CH_3_ or the addition of other groups) would be required to help in obtaining E9 P/T crystals. We are currently pursuing ways to improve binding.

Finally, it is notable that E9 is the sixth polymerase (three RTs [2,3] and three DNA polymerases [7]) to demonstrate strong sequence binding preferences. Of the previous five, the three RTs revealed a biologically significant preference for binding PPT-like sequence, while *Taq* and Klenow revealed a possible evolutionary/biochemical relationship between phage RNA polymerase-promoter recognition and bacterial DNA polymerases. As DNA polymerases generally recognize recessed 3′ termini for binding and activity without preferred sequence context, the role of sequence preferences for DNA polymerases is unclear, and preferred binding is, perhaps, unexpected. However, the examples noted above indicate that these enzymes do, in fact, have sequence preferences, and they can potentially be exploited for structural analysis, as well as for understanding their evolutionary and biochemical relationships with other DNA-binding proteins.

## Figures and Tables

**Table 1:** Apparent equilibrium dissociation (K_D,app_), off-rate (*k*_off_) and half-life (t_1/2_) values for selected sequences.

Sequence Name ^1^	K_D,app_ (nM) ^2^	*k*_off_ (min^−1^)	t_1/2_ (min) ^3^
Control ^4^ 5′---ATCATGGTAGCGTCGATAATT-3′ 3′---TAGTACCATCGCAGCTATTAAGCGT-5′	UTD ^5^	ND ^6^	ND
E9-R5-3 5′---AGGCAGAGACGCCGTGACCTC-3′ 3′---TCCGTCTCTGCGGCACTGGAGGTCC-5′	UTD	ND	ND
E9-R5-4 5′---GTAATACCCCTAACCTACCAG-3′ 3′---CATTATGGGGATTGGATGGTCCCTC-5′	UTD	ND	ND
E9-R5-12 5′---CTGCCCATGGACACCCAACAG-3′ 3′---GACGGGTACCTGTGGGTTGTCCGTC-5′	93 ± 7	0.083 ± 0.019	8.6 ± 2.0
E9-R5-12 60 nt loop-back (see Figure 5)	ND	0.094 ± 0.020	7.6 ± 1.8
E9-R5-12 50 nt loop-back (see Figure 5)	ND	0.107 ± 0.039	7.1 ± 2.4
E9-R5-12 40 nt loop-back (see Figure 5)	ND	UTD	UTD
E9-R5-12 gacta-1 ^7^ 5′---CTGCCCATGGACACCCGACTA-3′ 3′---GACGGGTACCTGTGGGCTGATCGTC-5′	UTD	ND	ND
E9-R5-12 gacta-2 5′---CTGCCCATGGAGACTAAACAG-3′ 3′---GACGGGTACCTCTGATTTGTCCGTC-5′	UTD	ND	ND
E9-R5-12 gacta-3 5′---CTGCCCGACTACACCCAACAG-3′ 3′---GACGGGCTGATGTGGGTTGTCCGTC-5′	UTD	ND	ND
E9-R5-12 gacta-4 5′---CGACTAATGGACACCCAACAG-3′ 3′---GCTGATTACCTGTGGGTTGTCCGTC-5′	UTD	ND	ND

^1^—Sequences from PT SELEX are named according to the protein used for selection (E9), the SELEX round number and the particular sequence number. Nucleotides at the 5′ primer 3′ template regions (designated with ---) are not shown and were primer: 5′-GCCTGCAGGTCGACTCTAGA-3′; and template: 5′-TCTAGAGTCGACCTGCAGGC-3′. Sequences labeled as “loop-back” were single strands of DNA derived from the double stranded E9-R5-12 primer-template sequence by removing primer and template sequences from the 5′ end of the primer and 3′ end of the template and adding a 3-nt “TTT” loop. ^2^—All assays were conducted in buffer containing: 20 mM Tris-HCl pH 7.5, 90 mM KCl, 60 mM NaCl, 4 mM DTT, 0.1 µg/µL BSA, and 2% glycerol. For all data, values given are averages of 3 independent experiments ± S.D. ^3^—t_1/2_ was derived from the *k*_off_ value using the equation: t_1/2_ = 0.69/*k*_off_^4^—The control sequence was randomly selected from the starting pool from a previous PT SELEX experiment (DeStefano and Cristofaro, 2006, *Nucleic Acids Res.* 34(1):130–139). ^5^—“UTD”—Unable to determine. The value was not able to be determined using the assay conditions with the amount of protein used. ^6^—“ND”—Not determine. No attempt was made to determine the value. ^7^—Sequences were derived from E9-R5-12 by replacing a portion of the duplex with a 5′-GACTA-3′ sequence as indicated and underlined.

## Data Availability

Data are contained in the article or Supplemental Materials.

## Supplementary Materials

The following supporting information can be downloaded at: https://www.mdpi.com/article/10.3390/v14020369/s1, Figure S1. Dissociation of E9 vaccinia virus polymerase from P/T constructs select with E9 or other proteins (see Table 1) shows that only E9-R5-1 binds stably; Figure S2. Extension of selected radiolabeled primer-templates with E9 polymerase shows that all primer templates are extended in a similar fashion and the E9-R5-12 is not extended better than P/T constructs selected with other polymerases; and Table S1: Primer-template sequences selected by PT SELEX examined for binding and extension with vaccinia virus polymerase (E9).
