# Supplementary material for: Selection of Primer–Template Sequences That Bind with Enhanced Affinity to Vaccinia Virus E9 DNA Polymerase
Source: Viruses. 2022 Feb 10;14(2):369. doi: 10.3390/v14020369 (PMC8880465; doi:10.3390/v14020369)
Supplement: Supplementary file 1 [file viruses-14-00369-s001.zip › viruses-1493525-supplementary.pdf]

Supplementary Materials for:

Selection of Primer-Template Sequences that Bind with Enhanced Affinity to Vaccinia Virus E9 DNA Polymerase

by

Jeffrey J. DeStefano [1,2,*], Frédéric Iseni [3] and Nicolas Tarbouriech [4]

[1]Cell Biology and Molecular Genetics, Bioscience Research Building, University of Maryland, College Park, MD 20742 USA

[2]Maryland Pathogen Research Institute (MPRI), University of Maryland, College Park, MD 20742 USA

[3]Unité de Virologie, Institut de Recherche Biomédicale des Armées, BP73, F-91223 Brétigny-sur-Orge Cedex, France

[4]Institut de Biologie Structurale, Université Grenoble Alpes, CNRS, CEA, IBS, F-38000 Grenoble, France

*Correspondence: jdestefa@umd.edu; Tel: +1-301-405-5449

Methods

Comparing the dissociation of E9 polymerase from selected. Ten nM (final concentration) 5′-^{32}P end-labeled P/T was incubated for 10 min at room temperature in 72 µl of 50 nM E9 protein, 20 mM Tris-HCl pH 7.5, 90 mM KCl, 60 mM NaCl, 4 mM DTT, 0.1 µg/µl BSA, and 2% glycerol. The high concentration of E9 was required due to the sequences binding relatively weakly under these conditions. At time "0", 8 µl of heparin (10 µg/µl) in the same buffer was added to the solution. Ten µl aliquots were removed at 0.25, 1, 2, 4, 8, and 16 minutes and filtered over nitrocellulose which was washed with 3 ml of buffer (25 mM Tris-HCl, pH 7.5, 10 mM KCl). A background control was prepared by mixing 10 nM of the same 5′ ^{32}P end-labeled P/T being tested and 10 µg of heparin, then adding E9 protein (50 nM final concentration) in a total of 10 ul of buffer. This tests the effectiveness of heparin in "trapping" E9 protein and preventing its binding to P/T. This sample was incubated for 16 min before processing and was subtracted from the other samples in the final calculations. The percent of total P/T that remained bound to the filter was plotted vs. time to generate the graph (Fig. S1). Time "0" samples that were removed prior to the addition of heparin were also performed and indicated that all the P/Ts bound E9. These are not included in the graph as they were not repeatable with statistical accuracy for any samples except the E9 selected P/T. Despite binding the P/T, only the E9 selected P/T remained bound at a significant level at the 0.25-minute time point, which was the earliest point feasible for analysis after heparin addition.

Extension of P/T SELEX sequences with E9 polymerase. Reactions were performed under the same conditions as the dissociation rate constant determinations except that 25 nM of the primer-template sequences (see Table S1) labeled with ^{32}P at the 5′ end of the primer strand was used, and 2 mM $MgCl_2$, and 50 µM dNTPs were included. Reactions were initiated by adding E9 at 25, 50, 100, or 200 nM to a total volume of 10 µl and incubating at 30 ºC for 5 min. Reactions were terminated with 10 µl of 2X loading buffer (90% formamide, 10 mM EDTA pH 8, 0.025% bromophenol blue and xylene cyanol) and the samples were run on a 10% denaturing polyacrylamide gel. Material was visualized using a phosphorimager (Fuji FLA 7000).

Table S1. Primer-template sequences selected by PT SELEX examined for binding and extension with vaccinia virus polymerase (E9)

Primer Template (P/T) sequence*	Enzyme used for selection in PT SELEX
5’-gcctgcaggtcgactctagaCTGCCCATGGACACCCAACAG-3’(41)<br>3’-cggacgtccagctgagatctGACGGGTACCTGTGGGTTGTCCGTC-5’(45)	E9 (E9-R5-12 from main text)
5’-gcctgcaggtcgactctagaCCCAGTCCACACTAAAGCATA-3’(41)<br>3’-cggacgtccagctgagatctGGGTCAGGTGTGATTTCGTATCTGT-5’(45)	*Taq* (selected sequence 1)[1]
5’-gcctgcaggtcgactctagaCCCCAATTTGCGAACGTGCCT-3’(41)<br>3’-cggacgtccagctgagatctGGGTCAGGTGTGATTTCGTATCAGC-5’(45)	*Taq* (selected sequence 2)[1]
5’-gcctgcaggtcgactctagaAAACTGAAGAGGCACCGAAGGGGGGG-3’(46)<br>3’-cggacgtccagctgagatctTTTGACGGCGCCGTGGCGGCCCCCCCAGAA-5’(50)	HIV reverse transcriptase (HIV RT)[2]
5’-gcctgcaggtcgactctagaCAACCATCGAAGACTA-3’(36)<br>3’-cggacgtccagctgagatctGTTGGTAGCTTCTGATATCGTCCGT-5’(45)	Exonuclease minus Klenow (exo$^-$ Klenow)[1]

1-Fenstermacher et al., J. Bacteriol. 2018 Apr 1;200(7).
2-DeStefano and Cristofaro, Nucleic Acids Res. 2006;34(1):130-9.
*Note that the primer and template lengths (indicated in parentheses after the sequence) are not identical for all P/Ts used. For HIV RT P/T, PT SELEX was performed using a longer construct with a 30 nucleotide random region rather than 25. For exo$^-$ Klenow, analysis showed the enzyme bound only to constructs with a shortened version of the primer. See references above for more details. Nucleotides in bold were derived from the random region of the material used for selections while the small letters correspond to sequences from fixed regions of the starting construct.

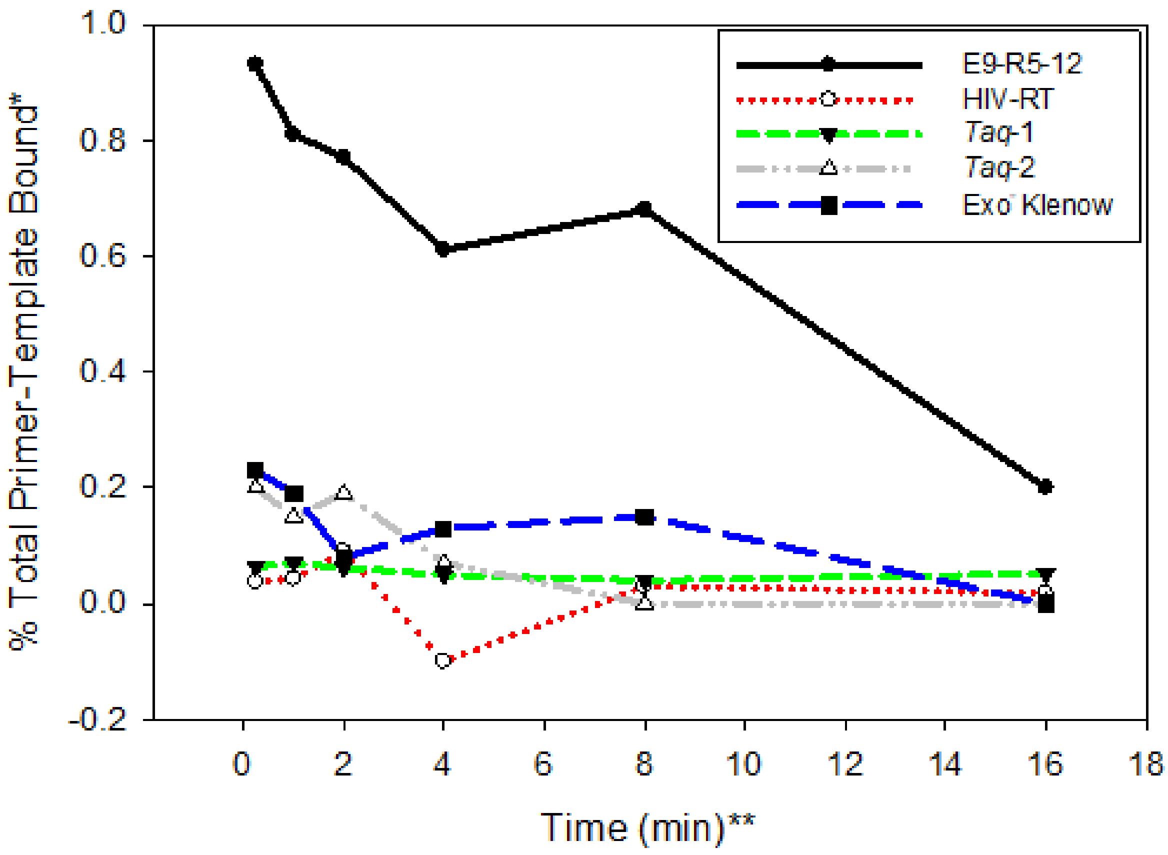

Figure S1. Dissociation of E9 vaccinia virus polymerase from P/T constructs select with E9 or other proteins (see Table 1) shows that only E9-R5-1 binds stably. E9 protein was mixed with P/T labeled with 32P as described in Methods. Dissociation in the presence of heparin "trap" (see Methods and main text) was measure by measuring the amount of P/T bound to E9 material at 0.25, 1, 2, 4, 8, and 16 min. *This is expressed as the % of the total P/T in the assay (10 nM) bound to E9 vs. Time. **Note that the first point shown for each P/T is the 0.25 min point rather than a time "0" (see Methods). Therefore, the assay does not indicate that E9 cannot bind the other P/Ts but that binding is essentially not observed or very low at 0.25 min. This experiment was repeated with similar results.

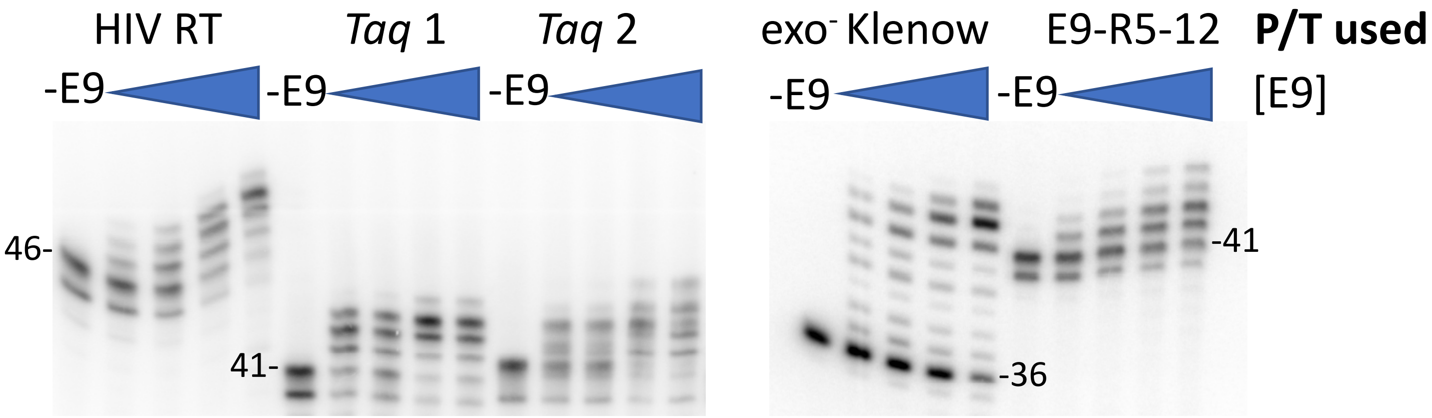

Figure S2. Extension of selected radiolabeled primer-templates with E9 polymerase shows that all primer templates are extended in a similar fashion and the E9-R5-12 is not extended better than P/T constructs selected with other polymerases. E9 protein (0, 25, 50, 100 or 200 nM) was mixed with 25 nM P/T labeled with ^{32}P at the 5' end of the primer strand and extension of the primer was allowed to continue for 5 min before terminating the reactions. The material was separated on a 10% polyacrylamide gel. The HIV RT, *Taq* 1 and *Taq* 2 samples are from one gel and the exo^{-} Klenow and E9-R5-12 samples from another. The sizes and positions of the non-extended primer strand are indicated (see Table S1 for details about the P/Ts used). "-E9", no E9 polymerase added to reactions. See Methods for details of the reactions. This experiment was repeated with similar results.
